# The development of the Metacognitions About Boredom Scale (MABS) and its role in problematic social media use

**DOI:** 10.1556/2006.2025.00499

**Published:** 2026-09-15

**Authors:** Mehdi Akbari, Roya Forootan, Zahra Tabar, Zsolt Demetrovics, Mark D. Griffiths

**Affiliations:** 1 Department of Clinical Psychology, Faculty of Psychology and Education, Kharazmi University, Tehran, Iran; 2 Department of Psychology, Faculty of Education and Psychology, Alzahra University, Tehran, Iran; 3 Flinders University Institute for Mental Health and Wellbeing, College of Education, Psychology and Social Work, Flinders University, Bedford Park, SA, Australia; 4 Institute of Psychology, ELTE Eotvos Lorand University, Budapest, Hungary; 5 Centre of Excellence in Responsible Gaming, University of Gibraltar, Gibraltar, Gibraltar; 6 Nottingham Institute of Psychology, Nottingham Trent University, Nottingham, UK

**Keywords:** internet use, social media use, online social networking, problematic technology use, psychometrics, metacognitions, boredom, addictive behaviors, problematic internet use, behavioral addictions

## Abstract

**Background and Aims:**

Boredom is a universal affective–cognitive state arising from unmet needs for meaningful engagement. Although unpleasant, boredom can inspire creativity and purposeful action or trigger maladaptive behaviors such as problematic social media use (PSMU). The present study developed and validated the Metacognitions About Boredom Scale (MABS), a new instrument designed to assess positive and negative metacognitions about boredom.

**Methods:**

The research comprised two studies. Study 1 involved mixed-methods item generation and large-scale validation among Iranian adults (*N* = 1,021; 58.0% females; mean age = 39.4 years), employing exploratory and confirmatory factor analyses. Study 2 involved at-risk individuals with problematic social media use (*N* = 234); bifactor and ROC analyses were conducted to determine an optimal cut-off score for positive and negative metacognitions about boredom.

**Results:**

Study 1 supported a stable two-factor structure of positive and negative metacognitions about boredom, confirmed through both exploratory and confirmatory analyses. Both subscales demonstrated excellent reliability, strong test–retest stability, and full measurement invariance across gender and age. Incremental validity analyses showed that the MABS predicted boredom and PSMU beyond general metacognitions. Study 2 confirmed the bifactor model, and ROC analysis indicated an optimal cut-off of ≥32 on the negative belief subscale for distinguishing individuals at risk of boredom-driven PSMU, yielding 95.3% sensitivity and 97.1% specificity.

**Conclusions:**

The MABS is a valid, theory-based tool assessing boredom-related metacognitions and identifying cognitive risks for maladaptive online behaviors.

## Introduction

### The nature and importance of boredom

Boredom is a universal phenomenon with intertwined emotional and cognitive components, occurring when an individual desires to engage in a rewarding activity but is unable to do so (Eastwood, Frischen, Fenske, & Smilek, 2012; Merrifield & Danckert, 2014). Emotionally, it arises in situations lacking motivation and challenge, leading to restlessness or disinterest. Cognitively, it is typically characterized by slowed time perception, difficulty concentrating, and the absence of meaningful goals (Akyıl, 2025; Ndetei, Nyamai, & Mutiso, 2023; Westgate & Steidle, 2020).

Although boredom is often seen as unpleasant, research shows its paradoxical nature with both positive and negative aspects: it can enhance creativity and innovation (Gasper & Middlewood, 2014; Mann & Cadman, 2014; Zeißig & Pannasch, 2023), but it is also associated with substance use, impulsivity, risky behaviors, and psychological harms (Bambrah, Wyman, Friedman, & Eastwood, 2022; Chao, Todman, Foltin, Evans, & Bedi, 2024; Matijašević, Mališa, & Vukašinović, 2025).

The literature on boredom as a multidimensional phenomenon highlights two perspectives. The adaptive view considers boredom a warning signal of meaningless or unworthy activity, directing the individual toward more rewarding or goal-oriented engagement (Bench & Lench, 2013; Bieleke, Ripper, Schüler, & Wolff, 2022; Westgate, 2020). In contrast, the vulnerability-focused view emphasizes its maladaptive role as a risk factor for adverse outcomes such as depression, anxiety, alexithymia, difficulties in emotion regulation and metacognitive functioning, and particularly problematic online behaviors (Iannattone, Mezzalira, Bottesi, Gatta, & Miscioscia, 2024; Tagliaferri et al., 2025; Zhao et al., 2024).

Recent studies have highlighted that individuals' beliefs about boredom, whether they are construed as an opportunity for reflection or as aversive states to be avoided, may critically determine whether boredom yields constructive or maladaptive outcomes (Danckert & Elpidorou, 2023; Tagliaferri et al., 2025; Westgate, 2020; Zeißig, 2023). Accordingly, this dual nature underscores the importance of understanding not only the immediate experience of boredom but also the beliefs individuals hold about it.

### Boredom through the lens of metacognitive theory

A comprehensive understanding of individuals' beliefs about boredom requires the consideration of metacognitive theory (Wells, 2000). Wells (2000, 2009, 2019), the founder of metacognitive theory, has suggested that the activation and maintenance of cognitive-affective processes (such as worry, rumination, and desire thinking) is dependent on stable beliefs individuals have about them, called metacognitive beliefs. According to this theory, human behavior relies more on these beliefs than on objective reality, which is a key transdiagnostic mechanism in disorders (such as obsessive compulsive disorder [OCD] and addictive behaviors) (Akbari et al., 2021, 2023, 2024a, 2024b, 2026a, 2026b; Bocci Benucci, Tonini, Roffo, Casale, & Fioravanti, 2024; Güneysu, Aslan, Batmaz, & Güneysu, 2025).

Metacognitive beliefs are usually divided into two types: (i) positive beliefs about the usefulness of cognitive-affective experiences and coping strategies (e.g., *“Worrying helps me prepare”*), which encourage their use (Cano-Lopez, Garcia-Sancho, Fernandez-Castilla, & Salguero, 2022; Kim et al., 2021; Salguero et al., 2021), and (ii) negative beliefs about the uncontrollability and danger of such experiences and strategies (e.g., *“My thoughts are uncontrollable and could harm me”*), which are associated with psychological difficulties and maladaptive coping such as anxiety, depression and addictive behaviors (Akbari, Zamani, Fioravanti, & Casale, 2021; Chen et al., 2021; Kim et al., 2021; Tagliaferri et al., 2025; Thingbak, Capobianco, Wells, & O'Toole, 2024).

Although the metacognitive framework has been applied extensively to worry (Nordahl et al., 2023a, 2023b), rumination (Cano-Lopez et al., 2022; Salguero et al., 2021), and other affective processes, little is known about its relevance to boredom. In support of this, research has shown that it is not boredom itself that drives maladaptive engagement, but rather the interpretive and regulatory strategies individuals employ to manage their experience of boredom (Bocci Benucci et al., 2024; Tagliaferri et al., 2025; Wegmann, Ostendorf, & Brand, 2018). In the present study, maladaptive engagement refers specifically to excessive and dysregulated forms of online behavior, particularly problematic social media use (PSMU), rather than adaptive or recreational digital engagement. Individuals may hold positive metacognitive beliefs about boredom, viewing it as a useful motivational signal that encourages self-reflection or goal pursuit and elevates prosocial tendencies (Bench & Lench, 2019; Elpidorou, 2018; Morse, Fine, & Friedlander, 2021; Van Tilburg & Igou, 2017). Conversely, negative metacognitive beliefs may frame boredom as intolerable, uncontrollable, or inherently harmful, potentially exacerbating avoidance behaviors and maladaptive coping (Ford & Gross, 2018; Havermans, Vancleef, Kalamatianos, & Nederkoorn, 2015; Nederkoorn, Vancleef, Wilkenhöner, Claes, & Havermans, 2016; Weybright, Caldwell, & Smith, 2015; Wilson et al., 2014).

Theoretically, such beliefs could explain individual variability in boredom responses and why some individuals transform boredom into a creative force while others become trapped in cycles of disengagement and problematic behaviors. Extending the metacognitive model to boredom therefore provides a novel lens for understanding its functional and dysfunctional pathways.

### Boredom and addictive behaviors

Boredom has been increasingly recognized as a transdiagnostic vulnerability factor that contributes to the onset and maintenance of various addictive behaviors. Empirical studies demonstrate that boredom proneness is strongly associated with both substance-related and behavioral addictions.

*Boredom and problematic internet use (internet, social media, smartphones, gaming):* Large systematic reviews and meta-analyses have reported that boredom is robustly associated with problematic smartphone use, problematic internet use, PSMU, as well as clinically recognized conditions such as gaming disorder (Camerini, Morlino, & Marciano, 2023; Tagliaferri et al., 2025; Zhao et al., 2024). Longitudinal data indicate that boredom proneness mediates the effect of adverse life events on increases in problematic smartphone use among undergraduates (Yang, Zhou, Liu, & Fan, 2023). Cross-sectional and longitudinal studies indicate that individuals with higher trait boredom are more prone to problematic internet and social media use, primarily because chronic boredom diminishes self-regulatory capacity and heightens attentional bias toward socially rewarding online cues (Cannito et al., 2023; Zhang, Wang, Xu, & Fu, 2023). Associations between boredom proneness and problematic internet use have also been documented among clinical and at-risk samples (e.g., adolescents with ADHD), suggesting the relationship holds across diverse populations (Chou, Chang, & Yen, 2018).

*Boredom and gambling:* Pathological gamblers score significantly higher on boredom proneness compared to non-gamblers, suggesting that gambling may serve as a maladaptive strategy to escape under-stimulation (Mercer & Eastwood, 2010). During the COVID-19 lockdown, boredom among male students was associated with greater engagement in sports betting and online gambling (Donati, Guido, De Meo, & Primi, 2021; Wang, Won, & Jeon, 2022). Moreover, leisure boredom has been identified as a predictor of gambling motivation (Emir, Özcan, & Aydin, 2025).

*Boredom and substance use (alcohol, tobacco, cannabis, other drugs):* Susceptibility to boredom is associated with increased alcohol and tobacco use among college students (Weybright et al., 2015). In treatment contexts, individuals with substance use disorders describe boredom as a major trigger for relapse, particularly when lacking structured activities or meaningful engagement (Iso-Ahola & Crowley, 1991). There is also evidence obtained in an experimental setting that boredom contributes to the possibility of self-administering nicotine or alcohol as a form of stimulation (Mercer-Lynn, Flora, Fahlman, & Eastwood, 2013).

Overall, boredom appears to be a transdiagnostic driver of addictive behaviors, functioning both as a precursor and as a maintenance factor across domains. Its role spans problematic online behaviors, problem gambling, substance abuse, and other compulsive behaviors, highlighting the need to conceptualize boredom within metacognitive frameworks and to address it explicitly in prevention and intervention strategies. Taken together, boredom emerges as a complex and multifaceted construct that can function as both an adaptive signal and a vulnerability factor, depending on how it is interpreted and regulated.

### Boredom, metacognition, and problematic social media use

Although PSMU has been widely examined in the literature, it is not formally recognized as a clinical disorder in diagnostic manuals such as the latest versions of the *Diagnostic and Statistical Manual of Mental Disorders* (DSM-5) and *International Classification of Diseases* (ICD-11). Accordingly, it is more appropriate to conceptualize PSMU as a maladaptive pattern of behavior characterized by excessive and dysregulated use, rather than a diagnosable condition. While widespread smartphone ownership (approximately 78% globally) has substantially increased the availability and convenience of social media platforms (e.g., *Facebook, Instagram, TikTok*), ownership alone does not sufficiently explain engagement patterns or problematic use (Dixon, 2022; O'Dea, 2021).

Engagement with social media is instead driven by heterogeneous and goal-directed motivations, including social connection, entertainment, information seeking, and –critically– the regulation of affective states. Among these, boredom has been increasingly identified as a salient affective trigger for social media engagement. Individuals experiencing boredom may turn to social media as an easily accessible and immediately rewarding means of alleviating understimulation or aversive internal states. Consistent with this view, empirical studies have demonstrated robust associations between boredom and PSMU (Bocci Benucci et al., 2024; Donati, Beccari, & Primi, 2022; Pi, Wang, Zou, Mo, & Guo, 2024; Tagliaferri et al., 2025).

From a theoretical perspective, the compensatory internet use theory posits that individuals may engage in online activities, including social media use, to cope with negative emotions and unmet psychological needs (Kardefelt-Winther, 2014). Within this framework, metacognitive beliefs about boredom play a crucial role. Individuals who perceive boredom as intolerable, uncontrollable, or harmful may experience heightened negative affect, increasing the likelihood of relying on social media as a maladaptive coping strategy, thereby fostering PSMU (Akbari & Griffiths, 2026; Camerini et al., 2023).

Crucially, emerging evidence suggests that the relationship between boredom and PSMU is not unidirectional but potentially reciprocal. While boredom can motivate increased engagement in social media use (boredom → use), prolonged and excessive use may, over time, impair attentional control and diminish reward sensitivity, thereby exacerbating vulnerability to future boredom (use → increased boredom). This bidirectional pathway highlights a self-reinforcing cycle in which boredom and PSMU mutually maintain and intensify one another (Brand et al., 2025; Tam & Inzlicht, 2024).

Also, the role of positive metacognitive beliefs (Elpidorou, 2018; Lomas et al., 2017), in this process, is that they may initiate boredom, but what prevents the cessation of boredom is negative metacognitive beliefs, which can lead individuals to engage more in addictive behaviors as a coping strategy (Kardefelt-Winther, 2014). Therefore, it is not simply the experience of boredom that causes problematic behavior, but rather mediators and moderators such as metacognitive beliefs and strategies. Among various forms of online engagement, PSMU represents a particularly relevant context due to its high prevalence, wide accessibility, and strong association with affect regulation processes. Social media platforms provide immediate and continuous opportunities for mood modification, which may make them especially responsive to boredom-related mechanisms (Andreassen, 2016; Brand, Young, Laier, Wölfling, & Potenza, 2019; Kardefelt-Winther, 2014; Kuss & Griffiths, 2017).

### The need for a specialized instrument to assess metacognitive beliefs about boredom

Considering the aforementioned literature, to address boredom within a psychopathological framework, it is essential to conceptualize it through the metacognitive lens. A first step in this direction may involve identifying individuals' positive and negative beliefs about boredom experiences. Developing a metacognitive psychometric instrument specifically related to boredom could facilitate this process, enabling researchers and clinicians to clarify the role of boredom in the onset and maintenance of its psychopathological impact. Such a measure would also help to elucidate how boredom contributes to maladaptive behaviors, including addictive tendencies, positioning it as a key emotion implicated in both dysfunction and potential resilience pathways.

Research indicates that most current psychometric instruments have primarily focused on the trait aspects of boredom across various domains (Fahlman, Mercer-Lynn, Flora, & Eastwood, 2013; Farmer & Sundberg, 1986; Harasymchuk & Fehr, 2012; Nett, Goetz, & Daniels, 2010; Reijseger et al., 2013). Apart from initial efforts to develop the Boredom Beliefs Scale (BBS; Tam, Chan, van Tilburg, Lavi, & Lau, 2023), there are still no widely validated instruments for the direct assessment of metacognitive beliefs about boredom.

Given this limitation, and based on research showing that the positive and negative outcomes of boredom largely depend on how individuals interpret and respond to this emotion (Gross, 2015; Wells, 2000), there is a clear need for an instrument capable of assessing the two dimensions of boredom from a metacognitive perspective. Therefore, the aim of the present study was to design and validate a measure assessing positive and negative metacognitions related to boredom, and to examine whether this scale, in addition to predicting boredom and PSMU (as a potential outcome resulting from metacognitions related to boredom), could provide valid cut-off points for identifying individuals at risk for PSMU. A stable two-factor structure, differentiating between positive and negative metacognitions, was expected. Moreover, the two subscales were anticipated to show distinct associations with adaptive and maladaptive traits: positive metacognitions aligning with openness, cognitive reappraisal, and self-efficacy, and negative metacognitions aligning with neuroticism, suppression, and PSMU. The predictive ability and the determination of cut-off points are highly important, as they indicate that the instrument is both scientifically valid and reliable, as well as being practically useful in clinical settings.

## Methods (study 1): the development of the metacognitions about boredom scale

### Participants and sampling

The present study utilized a large community sample of Iranian adults (*N* = 1,021; 58.0% females; Table 1). Participants' ages ranged from 18 to 70 years, with a mean age of 39.4 years (SD = 17.32). Participants were recruited via a multi-source convenience sampling strategy using internet platforms (e.g., social media platforms, online advertisements, and professional mailing lists) to enhance demographic diversity across age, education, and occupational groups. The gender distribution was approximately balanced (58.0% females, 42.0% males), ensuring representativeness across demographic subgroups. For structural validation, the total sample (*N* = 1,021) was randomly divided into two independent subsamples using R's random number generator (R Core Team, 2023; set.seed = 2024). The first sample was used for the exploratory factor analysis (EFA; *n* = 521) and the other was used for confirmatory factor analysis (CFA; *n* = 500). In terms of education, 19.3% of participants had less than a high school diploma, 33.6% had a diploma, 33.3% had an advanced diploma, 9.5% had a bachelor's degree, and 4.1% had a master's degree. With respect to occupational status, 41.2% were unemployed, 40.0% employed, and 18.7% were university students. Regarding marital status, 58.1% were single, 35.1% married, and 6.6% divorced. Participants also reported their daily social media use: 36.8% spent about one hour per day, 38.4% about 2 h, 16.8% about 3 h, 5.1% about 4 h, and 2.8% more than 4 h. Participants were eligible if they were aged 18 years or older, able to read Persian, and willing to provide informed consent. The study adhered to the Declaration of Helsinki, and was approved by the Institutional Review Board of Kharazmi University.

**Table 1.** Demographic features of the participants (*N* = 1,021)

Variable	*M*	*SD*
*Age (18–70 years)*	39.40	17.32
	*n*	%
***Gender***		
Female	592	58.0
Male	429	42.0
***Educational attainment***		
Lower than diploma	198	19.3
Diploma	344	33.6
Advanced diploma	339	33.3
Bachelor	98	9.5
Master	42	4.1
***Occupational status***		
Unemployed	421	41.2
Employed	409	40.0
University Student	191	18.7
***Marital status***		
Single	594	58.1
Married	359	35.1
Divorced	68	6.6
***Social media use/per day***		
1 h or less	376	36.8
2 h	392	38.4
3 h	172	16.8
4 h	52	5.1
More than 4 h	29	2.8

### Measures

#### Demographics questionnaire

The participants were asked about their age, gender, educational level, occupational status, and daily time spent on social media.

#### Big Five Inventory-10 item (BFI-10)

The 10-item BFI-10 (Rammstedt & John, 2007; Persian version: Mohammad Zadeh & Najafi, 2010) was used to assess the Big Five personality traits (extroversion, agreeableness, conscientiousness, neuroticism, and openness). It is the shortest valid version of the Big Five Inventory (BFI; Rammstedt & John, 2007). Each item is rated on a five-point scale from 1 (*completely disagree*) to 5 (*completely agree*). Higher scores indicate greater levels of a given personality trait. In the present study, the Cronbach's alphas of the five factors ranged from 0.69 to 0.75.

#### Metacognitive questionnaire (MCQ-30)

The 30-item MCQ-30 (Wells & Cartwright-Hatton, 2004; Persian version: Mohammadkhani & Farjad, 2009) was used to assess five metacognitions: positive beliefs about worry (e.g., *“Worrying helps avoid future problems”*), negative beliefs about uncontrollability and danger of worry (e.g., *“My worrying is dangerous”*), cognitive confidence (e.g., *“My memory can mislead me”*), need to control thoughts (e.g., “*I should be in control of my thoughts all of the time*”), and cognitive self-consciousness (e.g., *“I should control my thoughts all of the time”*). Each item is rated on a four-point scale from 1 (*do not agree*) to 4 (*agree very much*). Higher scores indicate a higher level of metacognition. In the present study, positive beliefs about worry and negative beliefs about uncontrollability and the danger of worry were used as indicators of metacognition. In the present study, the Cronbach's alphas for the two factors were 0.81 (positive beliefs about worry) and 0.78 (negative beliefs about uncontrollability and the danger of worry).

#### The Barratt Impulsiveness Scale–short form (BIS-15)

The 15-item BIS-15 (Spinella, 2007; Persian version: Javid, Mohammadi, & Rahimi, 2012) is a short form of the Barratt Impulsiveness Scale–11 (BIS-11; Patton, Stanford, & Barratt, 1995) and was used to assess three dimensions of trait impulsivity: motor, non-planning, and attentional impulsivity. Items are rated on a four-point scale from 1 (*rarely/never*) to 4 (*almost always/always*). Higher scores indicate higher impulsivity. In the present study, the Cronbach's alpha was 0.78.

#### Depression anxiety and stress scale, short form (DASS-9)

The DASS-9 (Persian version: Yusoff, 2013 – adaptation based on the validated DASS-21 Persian version by Sahebi et al. [2005]) is a brief measure derived from the original 21-item DASS (Lovibond & Lovibond, 1995) that assesses symptoms of depression (e.g*., “I felt that I had nothing to look forward to”*), anxiety (e.g., *“I tended to overreact to situations”*), and stress (e.g., *“I experienced trembling”*) using three items per subscale. The Persian version was adapted from the established DASS-21 Persian translation by selecting the corresponding items proposed by Yusoff (2013) to form the 9-item short version. Participants rated each item on a 4-point scale from 0 (“*did not apply to me at all*”) to 3 (“*applied to me very much or most of the time*”). In the present study, Cronbach's alphas for the total scale and subscales (depression, anxiety, and stress) were 0.84, 0.75, 0.73, and 0.70, respectively.

#### Bergen Social Media Addiction Scale (BSMAS)

The six-item BSMAS (Andreassen et al., 2016; Persian version: Lin, Broström, Nilsen, Griffiths, & Pakpour, 2017) was used to assess problematic social media use. Items (e.g., *“How often during the last year have you felt an urge to use social media more and more?”*) are rated on a five-point scale from 1 (*very rarely*) to 5 (*very often*). A score above 24 indicates a high risk for PSMU (Luo et al., 2021). In the present study, the Cronbach's alpha was 0.76.

#### Metacognitions about social media use scale (MSMUS)

The 12-item MSMUS (Akbari et al., 2023 [originally developed in Persian]) was used to assess metacognitive beliefs regarding social media use. Items are rated on a four-point scale from 1 (*do not agree*) to 4 (*agree very much*) and comprises two subscales: positive metacognitions (e.g., *“Using social media reduces my negative feelings”*) and negative metacognitions (e.g., *“I continue to use social media despite thinking it would be better to stop”*). In the present study, the Cronbach's alpha for positive metacognitions was 0.96 and for negative metacognitions was .89.

#### The brief Sensation Seeking Scale (BSSS)

The eight-item Brief Sensation Seeking Scale (BSSS; Hoyle et al., 2002), derived from the original Sensation Seeking Scale (Zuckerman et al., 1964), was used to assess sensation-seeking behaviors. The scale comprises four subscales (experience seeking, boredom susceptibility, thrill and adventure seeking, and disinhibition) with two items each. Items (e.g., *“I would like to explore strange places”*) are rated on a five-point scale ranging from 1 (*strongly disagree*) to 5 (*strongly agree*). A higher score indicates greater sensation seeking. Because the BSSS has not been validated in the Persian language, the English version of the BSSS was translated into Persian by the Iranian authors and a bilingual psychologist following international translation guidelines (Beaton, Bombardier, Guillemin, & Ferraz, 2000). In the present study the Cronbach's alpha was 0.76.

#### Boredom Proneness Scale-short form (BPS-SF)

The eight-item BPS-SF (Struk, Carriere, Cheyne, & Danckert, 2017), derived from the original 28-item Boredom Proneness Scale (Farmer & Sundberg, 1986), was used to assess trait boredom. Items (e.g., *“Much of the time, I just sit around doing nothing”*) are rated on a seven-point scale from 1 (*strongly disagree*) to 7 (*strongly agree*). In the present study, the Cronbach's alpha was 0.88.

#### Emotion regulation questionnaire (ERQ)

The ten-item ERQ (Gross & John, 2003; Persian version: Foroughi et al., 2021) was used to assess emotion regulation. Items are rated on a seven-point scale from 1 (*strongly disagree*) to 7 (*strongly agree*) and there are two opposing dimensions. Six items assess the rate of cognitive reappraisal (e.g., *“I control my emotions by changing the way I think about the situation I'm in”*) and four items assess the rate of expressive suppression (e.g., *“When I am feeling negative emotions, I make sure not to express them”*). In the present study, the Cronbach's alphas for total score, cognitive reappraisal, and expressive suppression were 0.73, 0.76, and 0.75, respectively.

#### General self-efficacy scale (GSE)

The 10-item GSE (Schwarzer & Jerusalem, 1995; Persian version: Delavar & Najafi, 2013) was used to assess a broad and stable sense of personal competence to effectively deal with a variety of stressful life demands. Items (e.g., *“I can always manage to solve difficult problems if I try hard enough”*) are rated on a four-point scale ranging from 1 (*not at all true*) to 4 (*exactly true*). Higher scores indicate greater perceived self-efficacy. In the present study, the Cronbach's alpha was 0.89.

## Metacognitions about boredom scale (MABS) development

### Step 1: item generation and thematic categorization for MABS

The development of the MABS began with a qualitative exploration to identify the range of beliefs individuals hold regarding boredom. Face-to-face interviews were conducted with 41 participants (mean age = 36.1 years, SD = 14.9; 56% female) recruited from the general community. Guiding questions were designed to capture two complementary perspectives: (i) *general beliefs about boredom*: participants were asked about their overall views and attitudes toward boredom, for example, whether they perceived it as beneficial, harmful, or neutral in everyday life; and (ii) *situational experiences of boredom:* participants were asked to recall specific instances when they felt bored and described their thoughts, feelings, and behaviors in those moments. This dual approach ensured that item generation encompassed both abstract cognitive beliefs and concrete experiential patterns, strengthening the construct validity of the scale. For detailed information on thematic analysis and item extraction, see Appendix B. This process resulted in a pool of 30 candidate items covering both positive and negative metacognitive beliefs about boredom, which were then prepared for expert review.

### Step 2: expert review and content validity

A panel of 10 experts in clinical psychology, metacognitive therapy, and psychometrics evaluated the preliminary items for clarity, relevance, cultural appropriateness, and representativeness. Items with low Content Validity Index (CVI <0.78) or Content Validity Ratio (CVR <0.62) were revised or removed (Lawshe, 1975; Polit & Beck, 2006). Based on expert feedback, 16 items were retained—eight representing positive and eight representing negative metacognitive beliefs about boredom. These items constituted the preliminary version of the MABS, ready for subsequent psychometric testing.

### Data analysis

Data analysis was conducted in R (via Jamovi 2.3.28) using multiple specialized packages to evaluate the psychometric properties of the scale.

#### Item analysis and reliability

Item-level descriptive statistics (mean, standard deviation, skewness, kurtosis) and corrected item–total correlations were calculated to evaluate item performance. Internal consistency and reliability indices were examined using Cronbach's alpha, McDonald's omega, and Composite Reliability (CR). Additionally, person separation reliability was estimated within an Item Response Theory (IRT) framework using the eRm package (Mair, Hatzinger, Maier, Rusch, & Debelak, 2021). These analyses were intended to ensure the stability and internal coherence of each subscale prior to factor modeling.

#### Exploratory factor analysis (EFA) and Item Response Theory (IRT)

An Exploratory Factor Analysis was conducted using principal axis factoring with parallel analysis to determine the number of underlying factors. Promax (oblique) rotation was employed to allow for correlated latent constructs. Sampling adequacy and sphericity assumptions were tested prior to extraction. The EFA was designed to identify the latent structure underlying metacognitive beliefs about boredom and to examine whether items clustered into theoretically expected positive and negative factors. In addition, an Item Response Theory (IRT) approach based on the Partial Credit Model (PCM) was planned to assess item-level fit indices (infit and outfit statistics) and potential multidimensionality. The analysis framework allowed for the possibility that these two factors might be intercorrelated or represent higher-order latent processes.

#### Rasch model analysis

Following CFA, a series of Rasch model analyses were performed using the partial credit model (PCM) framework to further examine the scale's measurement properties. Within the Rasch framework, item difficulty, person ability, and fit statistics (infit and outfit mean squares) were inspected to evaluate item–person correspondence. Response category thresholds were analyzed to verify the ordering of Likert-scale response options. Items displaying misfit or disordered thresholds were flagged for potential revision.

#### Confirmatory factor analysis (CFA)

To cross-validate the factor structure identified in the EFA, a confirmatory factor analysis was conducted on an independent subsample using the robust weighted least squares estimator (WLSMV) implemented in the lavaan package (Rosseel, 2012). Bootstrapping with 5,000 replications was applied to obtain robust standard errors and confidence intervals. Two competing models—a unidimensional model and a two-factor model representing positive and negative metacognitive beliefs—were compared. Model fit was evaluated using conventional indices (CFI, TLI, RMSEA, SRMR) based on established cutoffs (Hu & Bentler, 1999). Standardized loadings and factor correlations were examined to confirm discriminant validity between the two belief dimensions.

#### Convergent and discriminant validity

Convergent and discriminant validity were examined by correlating the two MABS factors with theoretically relevant constructs, including adaptive and maladaptive cognitive–emotional strategies, metacognitive beliefs, personality traits, and self-regulatory indices. Average Variance Extracted (AVE) and inter-factor correlations were computed to test the Fornell–Larcker criterion for discriminant validity.

#### Incremental validity

Incremental validity was evaluated using hierarchical multiple regression analyses. Demographic and emotional variables were entered in the first step, followed by general metacognitions, domain-specific metacognitions, and finally the MABS subscales, to assess the unique contribution of boredom-related metacognitions beyond established predictors. All continuous predictors were mean-centered prior to entry, and multicollinearity was checked using variance inflation factors (VIFs <5). All model assumptions were verified using standard diagnostic checks and 5,000 bootstrap iterations.

#### Measurement invariance

Multi-group CFA was conducted to examine configural, metric, and scalar invariance across key demographic groups (gender and age). Invariance testing followed the sequential model-comparison approach to determine whether the factor structure, loadings, and thresholds were comparable across subgroups, ensuring the validity of cross-group comparisons.

### Ethics

This study was approved by the Institutional Review Board of Kharazmi University and conducted in accordance with the Declaration of Helsinki (2013 revision). Informed consent was obtained from all participants prior to their participation.

## Results

### Exploratory factor analysis

An EFA was conducted on a randomly selected subsample of *n* = 521 (Table 2). Sampling adequacy was confirmed (Kaiser–Meyer–Olkin = .91), and Bartlett's test of sphericity was significant, χ^2^ (120) = 2145.62, *p* < .001. Parallel analysis supported a two-factor solution, consistent with the expected structure. The two factors explained 54.2% of the variance (Factor 1 = 28.6%, Factor 2 = 25.6%). After Promax rotation, a clear and interpretable solution emerged: Items 1 to 8 loaded strongly on Factor 1 (Positive Metacognitions about Boredom [P-MABS]; loadings .69–.83). Items 9 to 16 loaded strongly on Factor 2 (Negative Metacognitions about Boredom [N-MABS]; loadings .72–.84). Cross-loadings were negligible (<|.20|), and communalities ranged from .25 to .69, indicating adequate shared variance. Overall, the EFA confirmed the hypothesized bidimensional structure of the scale.

**Table 2.** Exploratory factor analysis (*N* = 521)

Items No.	Factors loading		Uniqueness	Rasch model		
	Factor 1	Factor 2	1 - Communality	Mean	Infit	Outfit
1	**0.69**	−0.08	0.41	2.11	0.45	0.74
2	**0.70**	−0.06	0.35	2.78	0.76	0.62
3	**0.75**	0.03	0.30	3.39	0.53	0.88
4	**0.71**	0.02	0.27	3.43	0.80	0.77
5	**0.74**	0.05	0.49	3.68	0.88	0.79
6	**0.83**	0.08	0.39	3.83	0.65	0.84
7	**0.70**	0.03	0.53	3.98	0.83	0.99
8	**0.72**	−0.06	0.32	3.40	0.93	0.68
9	0.04	**0.76**	0.69	4.03	1.53	1.13
10	−0.15	**0.81**	0.25	4.13	1.55	1.37
11	−0.04	**0.80**	0.45	3.78	1.88	1.30
12	0.12	**0.78**	0.44	4.13	1.63	1.60
13	0.08	**0.79**	0.37	4.01	1.49	1.55
14	−0.19	**0.79**	0.39	3.80	1.64	1.64
15	0.08	**0.72**	0.46	3.79	1.67	1.68
16	−0.10	**0.84**	0.31	4.04	1.90	1.70

*Note*. Factor 1 (items 1–8): Positive Metacognitions About Boredom; Factor 2 (items 9–16): Negative Metacognitions About Boredom. Loadings ≥ .30 are in bold.

#### Rasch and IRT analyses

Complementary Rasch and Item Response Theory (IRT) analyses confirmed the psychometric adequacy of the scale. Items showed generally acceptable fit; however, threshold ordering was not uniform across all items, with several disordered adjacent response-category thresholds. Detailed Rasch and IRT results are presented in Appendix C.

### Confirmatory factor analysis

Confirmatory factor analysis was conducted on the second subsample (*N* = 500) to verify the latent structure identified in the exploratory phase. Results demonstrated that the hypothesized two-factor model—distinguishing positive and negative metacognitive beliefs about boredom—provided an excellent fit to the data and clearly outperformed a one-factor alternative. All standardized factor loadings were strong (.70–.88) and statistically significant, supporting the distinct yet correlated nature of the two belief dimensions. Model fit indices met established benchmarks, confirming the robustness and replicability of the two-factor structure.

Results strongly supported the two-factor solution. The two-factor model demonstrated excellent fit (CFI = .975, TLI = .971, RMSEA = .044, 90% CI [.039, .049], SRMR = .045), while the one-factor model exhibited poor fit (CFI = .841, TLI = .822, RMSEA = .105, SRMR = .098). The difference in fit between the two models was substantial (ΔCFI = .134), providing clear evidence in favor of distinguishing between positive and negative MABS. At the item level (see Table 3), all standardized factor loadings were strong, ranging from .70 to .84 for positive metacognitions and .71 to .88 for negative metacognitions. All loadings were statistically significant (*z*-values = 28.4–37.2, *p*-values < .001), and 95% confidence intervals did not cross zero, further supporting the robustness of the two-factor structure.

**Table 3.** Confirmatory factor analysis using robust diagonally weighted least-squares estimation (WLSMV; *N* = 500)

Observed	*β*	*LCI 95%*	*UCI 95%*	*z*	*p*
***Positive Metacognitions About Boredom***					
Item 1	0.77	0.72	0.78		
Item 2	0.81	0.75	0.84	29.4	< .001
Item 3	0.80	0.77	0.83	33.3	< .001
Item 4	0.71	0.70	0.79	28.4	< .001
Item 5	0.78	0.74	0.82	34.2	< .001
Item 6	0.79	0.72	0.81	30.5	< .001
Item 7	0.70	0.69	0.72	28.4	< .001
Item 8	0.84	0.81	0.88	31.5	< .001
***Negative Metacognitions About Boredom***					
Item 9	0.72	0.69	0.74		
Item 10	0.84	0.78	0.87	32.5	< .001
Item 11	0.81	0.74	0.83	29.4	< .001
Item 12	0.77	0.75	0.82	34.1	< .001
Item 13	0.79	0.76	0.84	30.9	< .001
Item 14	0.82	0.74	0.85	37.2	< .001
Item 15	0.71	0.70	0.79	28.8	< .001
Item 16	0.88	0.85	0.89	35.2	< .001

### Reliability and internal consistency

The reliability of the MABS was evaluated using both indices of internal consistency and test–retest stability. For the P-MABS subscale, Cronbach's alpha was .92, McDonald's omega was .90, and CR was .91. The N-MABS subscale demonstrated similar results, with a Cronbach's alpha of .94, McDonald's omega of .93, and CR of .92. All indices exceeded the recommended threshold of .70 (Nunnally & Bernstein, 1994), indicating excellent internal consistency and suggesting that the items within each subscale consistently assess the same latent construct.

To assess temporal stability, a subset of participants (*n* = 448) completed the scale twice over a two-week interval. Intraclass Correlation Coefficients (ICC; two-way mixed-effects model for absolute agreement, ICC [A,1]) indicated excellent stability, with values of .90 for the P-MABS and .93 for the N-MABS subscales, both surpassing the .70 benchmark for good test–retest reliability. Pearson product-moment correlations were similarly high (.89 for P-MABS and .92 for N-MABS), further supporting the stability of the scale scores. Paired-samples *t*-tests showed no significant mean differences between Time 1 and Time 2 for either subscale (P-MABS: *t* (447) = −1.12, *p* = .265; N-MABS: *t* (447) = 1.45, *p* = .149), indicating no systematic bias across administrations.

### Measurement invariance across gender and age

The results for gender-based invariance (male: *n* = 429; female: *n* = 592) are presented in Table 4. The configural model demonstrated acceptable fit, supporting the same baseline two-factor structure across gender. The metric invariance model showed negligible changes relative to the configural model (ΔCFI = −.001, ΔRMSEA = −.003, ΔSRMR = −.001). The scalar invariance model also remained within the prespecified criteria relative to the metric model (ΔCFI = .002, ΔRMSEA = .008, ΔSRMR = −.001), supporting scalar invariance across gender. Further constraints for scalar invariance did not worsen model fit (Δ*CFI* = 0.002, ΔRMSEA = −0.003, ΔSRMR = −0.001), supporting equivalence of item thresholds. Therefore, full scalar invariance was supported across gender, suggesting that observed score differences reflect true differences in the underlying latent constructs rather than measurement bias.

**Table 4.** Measurement invariance model using multi-group analysis (*N* = 1,021)

Model	*χ* ^2^	df	CFI	ΔCFI |≤0.010|	RMSEA	ΔRMSEA |≤ 0.015|	SRMR	ΔSRMR |≤0.030|	*p*-value	Invariant?
***The specified two-factor model***										
	924	192	0.975	–	0.044	–	0.045	–	< .001	–
**Multigroup factor analysis**										
***Gender (male vs. female)***										
Configural invariance	1,251	297	0.893	–	0.054	–	0.043	–	< .001	**Yes**
Metric invariance	1,292	299	0.892	−0.001	0.051	−0.003	0.042	−0.001	< .001	**Yes**
Scalar invariance	1,292	301	0.894	0.002	0.059	0.008	0.041	−0.001	< .001	**Yes**
***Age (young adults [18–29 years] vs. mid-age adults [30–40 years] vs. older adults [41 years and above])***										
Configural Invariance	1,178	423	0.891	–	0.059	–	0.041	–	< .001	**Yes**
Metric invariance	1,156	442	0.893	0.002	0.056	−0.003	0.052	0.011	< .001	**Yes**
Scalar invariance	1,143	498	0.892	−0.001	0.054	−0.002	0.058	0.006	< .001	**Yes**

The results for the three age groups are also presented in Table 4. For age, the configural model showed acceptable fit across young, middle-aged, and older adults. The metric invariance model showed changes of ΔCFI = .002, ΔRMSEA = −.003, and ΔSRMR = .011 relative to the configural model. The scalar invariance model showed changes of ΔCFI = −.001, ΔRMSEA = −.002, and ΔSRMR = .006 relative to the metric model. All absolute changes remained within the prespecified criteria, supporting scalar invariance across age groups. Similarly, the scalar invariance model demonstrated stable fit (ΔCFI = 0.001, ΔRMSEA = 0.001, ΔSRMR = 0.001), confirming that item thresholds were invariant. Together, the findings provided strong evidence of full measurement invariance across age, allowing valid comparisons of latent factor means among young, mid-aged, and older adults.

### Convergent and discriminant validity

To examine the convergent and discriminant validity of the MABS, correlations were computed between the scale's two subscales—P-MABS and N-MABS—and theoretically relevant constructs (Table 5). P-MABS showed significant positive correlations with adaptive cognitive-emotional strategies (e.g., cognitive reappraisal; *r* = .41, *p* < .001), positive beliefs about worry (*r* = .37, *p* < .001), and positive metacognitions about social media (*r* = .31, *p* < .001). P-MABS was also positively related to personality traits such as openness (*r* = .38, *p* < .001), extraversion (*r* = .35, *p* < .001), and conscientiousness (*r* = .29, *p* < .001), as well as sensation-seeking tendencies including experience seeking (*r* = .33, *p* < .001), thrill/adventure seeking (*r* = .39, *p* < .001), boredom susceptibility (*r* = .57, *p* < .001), and boredom proneness (*r* = .59, *p* < .001). Moreover, P-MABS was positively associated with general self-efficacy (*r* = .29, *p* < .001). In contrast, P-MABS was negatively related to maladaptive outcomes such as non-planning impulsivity (*r* = −.26, *p* < .01), attentional impulsivity (*r* = −.23, *p* < .01), motor impulsivity (*r* = −.25, *p* < .001), expressive suppression (*r* = −.18, *p* < .01), depression (*r* = −.19, *p* < .01), anxiety (*r* = −.22, *p* < .01), stress (*r* = −.25, *p* < .01), and PSMU (*r* = −.15, *p* < .01). These patterns suggest that P-MABS aligns with adaptive personality traits, effective cognitive-emotional regulation, and healthy novelty-seeking tendencies.

**Table 5.** Convergent and discriminant validity of the Metacognitions About Boredom Scale (*N* = 1,021)

Variable	Positive metacognitions about boredom	Negative metacognitions about boredom
**Metacognitions (MCQ-30)**		
Positive beliefs about worry	.37**	−.24**
Negative beliefs about worry	−.21**	.49**
Cognitive confidence	−.12*	−.22**
Need to control thoughts	.10*	.39**
Cognitive self-consciousness	.09	.10*
**Metacognitions about social media (MSMUS)**		
Positive metacognitions about social media	.31**	−.18*
Negative metacognitions about social media	−.19*	.39**
**Personality (BFI-10)**		
Openness to experience	.38**	−.14*
Extraversion	.35**	−.23*
Conscientiousness	.29**	−.08
Neuroticism	−.16*	.39**
Agreeableness	−.04	.07
**Emotion regulation (ERQ)**		
Cognitive reappraisal	.41**	−.21**
Expressive suppression	−.18*	.38**
**Impulsivity (BIS-15)**		
Motor impulsivity	−.25**	.38**
Attentional impulsivity	−.23**	.36**
Non-planning impulsivity	−.26**	.42**
**Emotional distress (DASS-9)**		
Depression	−.19*	.40**
Anxiety	−.22**	.44**
Stress	−.25**	.46**
**Problematic media use (BSMAS)**	−.15*	.45**
**Sensation seeking (BSSS)**		
Experience seeking	.33**	−.19*
Boredom susceptibility	.57**	−.28**
Thrill/adventure seeking	.39**	−.23*
**Boredom proneness (BPS-SF)**	.59**	−.29**
**General Self-Efficacy (GSE)**	.29**	−.36**

*Note*. *p < .01, **p < .001.

N-MABS was positively associated with maladaptive cognitive, emotional, and behavioral outcomes. More specifically, N-MABS showed moderate to strong positive correlations with negative beliefs about worry (*r* = .49, *p* < .001), need to control thoughts (*r* = .39, *p* < .001), neuroticism (*r* = .39, *p* < .001), cognitive self-consciousness (*r* = .10, *p* < .01), expressive suppression (*r* = .38, *p* < .001), impulsivity (motor *r* = .38, attentional *r* = .36, non-planning *r* = .42, all *p* < .001), and emotional distress including depression (*r* = .40, *p* < .001), anxiety (*r* = .44, *p* < .001), and stress (*r* = .46, *p* < .001). N-MABS was also positively related to negative metacognitions about social media (*r* = .39, *p* < .001) and PSMU (*r* = .45, *p* < .001). Conversely, N-MABS was negatively correlated with positive beliefs about worry (*r* = −.24, *p* < .001), cognitive confidence (*r* = −.22, *p* < .001), openness (*r* = −.14, *p* < .01), extraversion (*r* = −.23, *p* < .01), and general self-efficacy (*r* = −.36, *p* < .001). These findings highlight that maladaptive boredom-related metacognitions are strongly associated with emotional vulnerability, poor self-regulation, and problematic engagement in potentially maladaptive behaviors.

To further assess construct validity, the Average Variance Extracted (AVE) was computed. The AVE for the Positive Beliefs factor was .59, and for the Negative Beliefs factor it was .64, both exceeding the recommended cutoff of .50 (Fornell & Larcker, 1981). The correlation between the two latent factors was moderate (*r* = −.37), with the squared correlation (*φ*^2^ = .11) being smaller than the AVE values for both Positive (.59) and Negative (.64) factors. This finding satisfied the Fornell–Larcker criterion, suggesting adequate discriminant validity.

### Incremental validity of MABS in predicting boredom and problematic social media use

Table 6 presents the results of hierarchical regression analyses examining the incremental validity of the MABS in predicting boredom and PSMU. For boredom, demographic (gender, age), behavioral (daily hours of social media use), and emotional variables (anxiety, depression, stress) were entered at Step 1, explaining 11% of the variance. At Step 2, the five subscales of general metacognitions (MCQ-30) were added, accounting for an additional 7% of the variance (ΔR^2^ = .07). Step 3 included metacognitions about social media (MSMUS), which yielded a further 5% increase in explained variance (ΔR^2^ = .05). Finally, at Step 4, P-MABS and N-MABS were entered, resulting in the largest increment, explaining an additional 18% of the variance (ΔR^2^ = .18). Within this final step, both P-MABS (*β* = .39, *p* < .001) and N-MABS (*β* = .29, *p* < .001) emerged as robust predictors of boredom.

**Table 6.** Incremental validity of metacognitions about boredom in predicting boredom and problematic social media use: A hierarchical regression analysis (*N* = 1,021)

Step	Dependent variable	Predictor	*β*	*t*	*R* ^2^	ΔR^2^
**1**	**Boredom**	Gender	.05	2.10	0.11	–
		Age	−.13*	−5.90		
		Social media use (daily hours)	.18**	8.21		
		Anxiety	.17**	7.83		
		Depression	.15**	7.10		
		Stress	.11*	4.80		
**2**		Negative metacognitions about worry	.14*	5.50	0.18	**0.07**
		Positive metacognitions about worry	.10*	4.20		
		Need to control thoughts	.10*	4.50		
		Cognitive confidence	−.05	−2.10		
		Cognitive self-consciousness	.06	2.50		
**3**		Negative metacognitions about problematic social media use	.15**	7.31	0.23	**0.05**
		Positive metacognitions about problematic social media use	.11*	3.10		
**4**		Negative metacognitions about boredom	.29**	11.80	0.41	**0.18**
		Positive metacognitions about boredom	.39**	18.50		
**1**	**Problematic social media use**	Gender	.08	1.80	0.12	**—**
		Age	−.11*	−4.80		
		Social media use (daily hours)	.19**	8.00		
		Anxiety	.14*	7.50		
		Depression	.12*	4.40		
		Stress	.10*	4.10		
**2**		Negative Metacognitions about worry	.16**	6.80	0.18	**0.06**
		Positive Metacognitions about worry	.12*	4.80		
		Need to control thoughts	.11*	3.40		
		Cognitive confidence	−.04	−1.80		
		Cognitive self-consciousness	.05	2.30		
**3**		Negative metacognitions about problematic social media use	.22**	9.50	0.29	**0.11**
		Positive metacognitions about problematic social media use	.10*	3.90		
**4**		Negative metacognitions about boredom	.39**	17.10	0.50	**0.21**
		Positive metacognitions about boredom	.19**	9.20		

*Note.* ΔR^2^ indicates incremental variance explained at each step. *p < 0.05. **p < 0.001.

For PSMU, baseline predictors at Step 1 explained 12% of the variance. The inclusion of MCQ-30 subscales at Step 2 explained an additional 6% (ΔR^2^ = .06). At Step 3, MSMUS subscales accounted for a further 11% of the variance (ΔR^2^ = .11). In Step 4, P-MABS and N-MABS explained the largest share of incremental variance, with an additional 21% of the variance (ΔR^2^ = .21). Again, N-MABS (*β* = .39, *p* < .001) and P-MABS (*β* = .19, *p* < .001) were the strongest predictors of PSMU.

Taken together, these findings provided strong evidence for the incremental predictive validity of boredom-related metacognitions. The MABS explained substantial additional variance in both boredom and PSMU above and beyond demographic, emotional, general metacognitive, and domain-specific (social media) metacognitive factors. Importantly, both positive and negative MABS emerged as unique and powerful predictors, underscoring the central role of boredom-related metacognitions in maladaptive outcomes.

## Methods (study 2): validation and bifactor analysis of the MABS in an at-risk sample of social media users

### Participants and screening procedure for at-risk sample identification

A community sample of 234 individuals (mean age = 26.2 years, SD = 7.1; 56% male) at elevated risk for PSMU was recruited through public announcements at universities, counseling centers, psychology clinics, and social media platforms. Participants were initially screened using the Bergen Social Media Addiction Scale (BSMAS; Luo et al., 2021). Individuals scoring ≥24, a commonly used empirical threshold indicating clinically relevant levels of PSMU, were considered eligible for further assessment.

To improve classification accuracy, eligible participants completed a brief semi-structured clinical interview administered by trained clinical psychologists under the supervision of a licensed clinical psychologist. The interview assessed addiction-like symptoms and functional impairment across six domains derived from the addiction components model and DSM-5 criteria for behavioral addictions: salience, tolerance, mood modification, relapse, withdrawal, and conflict (American Psychiatric Association, 2013; Griffiths, 2005). Participants endorsing four or more domains were classified as being at risk for PSMU.

Participants were also required to report high social media use intensity (≥30 h per week) and at least one functional impairment associated with social media use (e.g., academic/occupational difficulties, sleep problems, interpersonal conflict, or emotional distress). Individuals with severe psychiatric disorders (e.g., schizophrenia, active bipolar disorder) or substance dependence were excluded.

Participants were identified through a multi-stage screening and verification procedure. In the first stage, volunteers aged 18–35 years completed an initial screening measure to identify individuals with elevated levels of problematic social media use. In the second stage, core addiction-related dimensions from the addiction components model (Griffiths, 2005)—including mood modification, conflict, withdrawal, and relapse—were assessed. Only individuals scoring high in at least two components progressed to the next stage. In the third stage, social media use intensity was evaluated. Inclusion criteria required ≥30 h per week of social media use, while recreational or low-frequency users (<1 h/day) were excluded. The fourth stage involved the assessment of functional impairment associated with social media use. Impairment was operationalized across multiple domains—including academic or occupational performance, sleep quality, interpersonal relationships, and emotional well-being—guided by criteria adapted from DSM-5 and aligned conceptually with ICD-11 indicators for behavioral addictions (World Health Organization, 2019). Participants reporting at least one substantial negative consequence advanced to the final stage.

In the fifth and final stage, eligible participants completed a semi-structured interview assessing addiction-like symptoms and functional impairment according to DSM-5 criteria for gambling disorder and internet gaming disorder. The interview covered six domains: salience, tolerance, mood modification, relapse, withdrawal, and conflict. Participants meeting four domains were classified as at-risk, whereas those meeting five or more domains were additionally categorized as high-risk, reflecting the diagnostically meaningful distinction in DSM-5 criteria. In the current sample, approximately 88% met exactly four domains (at-risk), and 12% met five or more domains (high-risk). For subsequent analyses, both groups were combined due to comparable metacognitive profiles. For the purposes of MABS validation, both subgroups were treated as part of the broader at-risk sample. Stages 3 and 5 were not directly compared. Stage 3 served primarily to quantify intensity of use, whereas Stage 5 provided a clinical verification of risk status and functional impact. Exclusion criteria included a history of severe psychiatric disorders (e.g., schizophrenia, active bipolar disorder) or substance dependence. All procedures adhered to the Declaration of Helsinki and were approved by the Institutional Review Board of Kharazmi University.

Regarding demographic characteristics, 44% of participants were female. Education levels ranged from less than a high school diploma (8%) to graduate-level degrees, with 18% holding a high school diploma, 11% an associate degree, and 41% a bachelor's degree. Most participants were single (63%), and 24% were students, while 37% were unemployed. On average, participants reported 34.2 h of social media use per week.

### Data integration

To evaluate structural validity across at-risk and community-based samples, data from the at-risk group were combined with the broader community sample of 1,021 participants from Study 1. This integration enabled a bifactor analysis to determine whether a boredom-related metacognition factor representing variance shared across all MABS items would overshadow the two specific metacognitive belief dimensions (positive and negative). The bifactor model comprised one general factor and two group-specific factors (positive and negative), allowing for the simultaneous assessment of both shared and distinct components of the construct.

### Data analysis

Model fit was evaluated using multiple goodness-of-fit indices, including the Comparative Fit Index (CFI), Tucker–Lewis Index (TLI), Root Mean Square Error of Approximation (RMSEA), and Standardized Root Mean Square Residual (SRMR). Omega Hierarchical (ωH) coefficients were computed to estimate the proportion of variance in subscale scores attributable to specific factors after accounting for the boredom-related metacognition factor. Factor loadings were inspected to ensure that each subscale retained meaningful and independent variance.

To assess the classification performance of the MABS subscales, receiver operating characteristic (ROC) curve analyses were conducted to distinguish participants in the prespecified at-risk PSMU group from community participants. The area under the curve (AUC) served as an index of the instrument's discriminative ability to distinguish between at-risk and non–at-risk users. Optimal cut-off points were determined using Youden's J-index optimization (Youden, 1950), maximizing the joint sensitivity and specificity for distinguishing at-risk from non–at-risk users. Additional classification indices—including sensitivity, specificity, positive predictive value (PPV), negative predictive value (NPV), overall accuracy, and Youden’s index—were calculated.

All inferential comparisons were adjusted for multiple testing using the False Discovery Rate (FDR) correction (Benjamini–Hochberg procedure, *Q* = .05). Finally, descriptive statistics and intercorrelations among subscales and general distress indicators were examined to support construct validity.

## Results

### Bifactor model fit and factor loadings

The bifactor model demonstrated a satisfactory overall fit: χ^2^ (89) = 451.33, CFI = .958, TLI = .942, RMSEA = .051 [.046, .056], SRMR = .044. Examination of standardized loadings showed that both the positive and negative subscales retained substantial independent variance even after controlling for the general factor. Omega hierarchical coefficients were .68 for the positive subscale and .89 for the negative subscale, exceeding the recommended threshold of .50, indicating that each subscale accounted for a meaningful proportion of reliable variance beyond the general psychopathology factor. The results supported the robustness and psychological relevance of the positive and negative metacognitive belief factors within an at-risk sample, confirming that both dimensions captured distinct yet complementary aspects of metacognitive functioning.

Although both groups were combined for subsequent analyses as a single at-risk profile, additional analyses were conducted to examine whether the at-risk and high-risk subgroups differed in risk classification and ROC performance as well as to ensure that combining at-risk and high-risk participants did not bias the validation results. N-MABS and P-MABS scores were compared between subgroups. No significant differences were observed (all *p-*values > .05), and multi-group confirmatory factor analyses confirmed invariance of the bifactor structure across subgroups. The discriminative performance of the N-MABS subscale was consistent across participants classified as at-risk vs. high-risk, supporting the robustness of the subscale across the risk spectrum. These findings justified treating both subgroups as a single at-risk sample for subsequent psychometric analyses.

### Risk classification and ROC analysis

Trapezoidal integration of the ROC operating points reported in Table 7 yielded an AUC of .977 for N-MABS (Hanley–McNeil 95% CI [.964, .991], p < .001), indicating excellent discrimination between participants in the prespecified at-risk PSMU group and community participants (Fig. 1). Among the reported thresholds, a cutoff of ≥32 produced the highest Youden’s index (92.4%) and overall accuracy (96.7%), with 95.3% sensitivity, 97.1% specificity, 88.1% positive predictive value (PPV), and 98.9% negative predictive value (NPV). Adjacent cutoffs of 31 and 33 also showed high accuracy (95.7% and 96.6%, respectively), but ≥32 provided the best overall balance of sensitivity and specificity.

**Table 7.** Receiver Operating Characteristic (ROC)–based cut-off scores for Negative and Positive MABS (*N* = 1,255; 234 at-risk, 1,021 community participants)

Cut-off	True positive	True negative	False positive	False negative	Sensitivity (%)	Specificity (%)	PPV (%)	NPV (%)	Accuracy (%)	Youden's index (%)
Negative metacognitions about boredom										
30	229	960	61	5	97.9	94.0	79.0	99.5	94.7	91.9
31	226	975	46	8	96.6	95.5	83.1	99.2	95.7	92.1
**32**	**223**	**991**	**30**	**11**	**95.3**	**97.1**	**88.1**	**98.9**	**96.7**	**92.4**
33	217	996	25	17	92.7	97.6	89.7	98.3	96.6	90.3
34	210	1,000	21	24	89.7	98.0	90.9	97.7	96.4	87.7
Positive metacognitions about boredom										
26	185	590	431	49	79.1	57.8	30.0	92.3	61.8	36.9
27	196	620	401	38	83.8	60.7	32.8	94.2	65.0	44.5
**28**	**205**	**650**	**371**	**29**	**87.6**	**63.7**	**35.6**	**95.7**	**67.9**	**51.3**
29	187	700	321	47	79.9	68.5	36.8	93.7	70.7	48.4
30	160	745	276	74	68.4	73.0	36.7	91.0	72.0	41.4

*Note*. Sensitivity = true positive/(true positive + false negative), specificity = true negative/(true negative + false positive); accuracy = (true positive + true negative)/N; PPV: positive predictive value = true positive/(true positive + false positive); NPV: negative predictive value = true negative/(true negative + false negative); Youden's index= sensitivity + specificity – 100. Emboldened rows represent optimal thresholds determined by ROC analysis. For N-MABS, scores greater than or equal to the cutoff were classified as positive; for P-MABS, scores less than or equal to the cutoff were classified as positive.

**Fig. 1.**
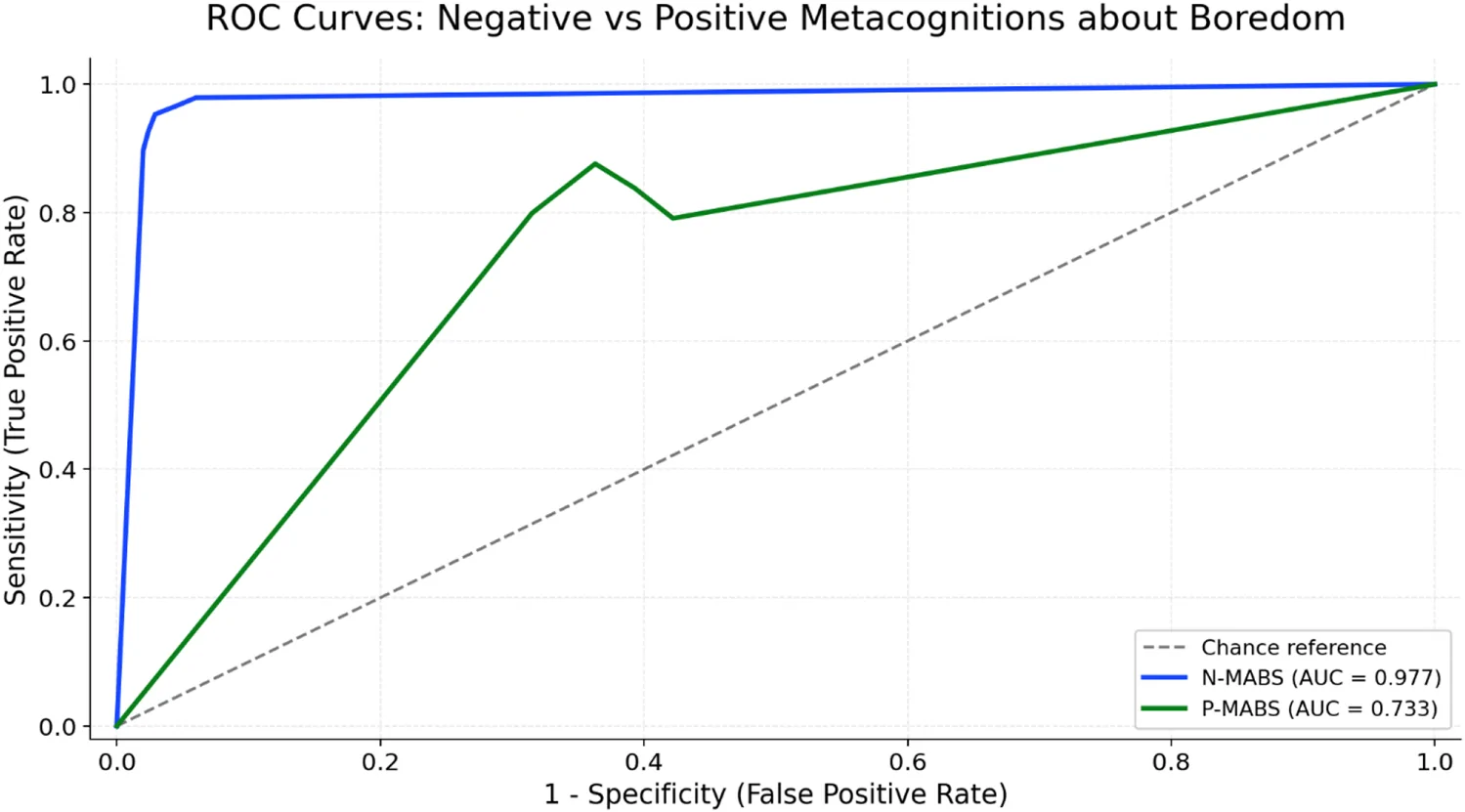
Receiver operating characteristic (ROC) curves for the Negative and Positive Metacognitions About Boredom Scale subscales in distinguishing the prespecified at-risk PSMU group from community participants

P-MABS demonstrated comparatively weaker classification performance, with an AUC of .733 (Hanley–McNeil 95% CI [.694, .772], p < .001; Fig. 1). Among the reported P-MABS thresholds, a cutoff of ≤28 yielded the highest sensitivity and Youden’s index (51.3%), with 87.6% sensitivity, 63.7% specificity, 35.6% PPV, 95.7% NPV, and 67.9% accuracy. Although cutoffs of 29 and 30 produced higher overall accuracy (70.7% and 72.0%, respectively), they had lower sensitivity and lower Youden’s indices, indicating a less balanced classification profile.

Scores above 28 were less characteristic of the prespecified at-risk group and may tentatively reflect relatively stronger positive or adaptive beliefs about boredom. Such beliefs could potentially have protective relevance in self-regulatory or therapeutic contexts; however, this possibility should be interpreted cautiously because it was not directly tested by the ROC analysis.

Overall, N-MABS—particularly at the ≥32 threshold—showed substantially stronger classification performance than P-MABS. The ≥32 cutoff may therefore be considered a research-based screening threshold for distinguishing the prespecified at-risk PSMU group from community participants, rather than a diagnostic cutoff. P-MABS provided more limited but potentially complementary information concerning positive or adaptive boredom-related metacognitions.

Although Fig. 1 primarily presents the ROC curves and their classification performance, the contrast between the two subscales may also be viewed as broadly consistent with the proposed conceptual framework in which negative and positive boredom-related metacognitions could contribute differently to maladaptive and potentially adaptive self-regulatory outcomes. These broader cognitive–affective interpretations remain theoretically informed and require direct examination in future longitudinal or experimental research.

## Discussion

### Summary of main findings

The present study developed and validated the MABS, confirming a stable two-factor structure—positive and negative beliefs about boredom—with strong reliability and validity indices. Beyond these psychometric results, the findings offer theoretical insight into how individuals think about and regulate boredom, and how these beliefs may underpin adaptive and maladaptive self-regulatory outcomes such as problematic social media use.

Importantly, a sample of 234 at-risk problematic social media users was used to determine a research-based threshold (≥32 for N-MABS) on the MABS, providing a valuable empirical benchmark for future studies and applied screening contexts. The bifactor model showed an acceptable fit among this sample, and even after controlling for general distress, both subscales accounted for significant unique variance. These results support the construct validity of the MABS and indicate that metacognitive beliefs about boredom represent a distinct psychological construct, separable from general distress and deserving independent consideration within theoretical frameworks of behavioral addictions. Importantly, the identified N-MABS threshold (≥32) should not be interpreted as a diagnostic cut-off equivalent to structured clinical interviews, but rather as a process-sensitive indicator of maladaptive metacognitive tendencies associated with risk. In contrast to symptom-based diagnostic frameworks (e.g., DSM-5 criteria for behavioral addictions), which focus on observable behaviors and functional impairment, the MABS captures higher-order cognitive appraisals about boredom that may precede and shape these symptoms.

From a clinical perspective, this positions the MABS as a complementary tool that provides incremental value beyond traditional assessments by identifying cognitive vulnerability mechanisms that are not directly observable in standard diagnostic interviews. Therefore, the MABS may enhance early detection and case formulation by indexing metacognitive processes that contribute to the emergence and persistence of PSMU.

### Comparison with previous research

Two widely used instruments in the study of boredom are the Boredom Proneness Scale (BPS; Farmer & Sundberg, 1986) and the Boredom Beliefs Scale (BBS; Tam et al., 2023). The BPS is a traditional instrument that conceptualizes boredom as a personality trait and assesses individuals' general tendency to experience boredom across different contexts. In contrast, the BBS focuses on evaluative beliefs about boredom (e.g., whether it is considered natural or undesirable), providing a clearer understanding of individual attitudes toward boredom. However, despite capturing both positive and negative dimensions of boredom, it does not address the metacognitive dimension, and its psychometric performance is limited because the original validation sample consisted primarily of adolescents and young adults.

The present study developed the MABS, which differs conceptually and methodologically from previous instruments and provides an incremental advancement in the assessment of boredom-related processes. This distinction operates across two complementary domains (i.e., theoretical and psychometric/applied). In the theoretical domain, the MABS moves beyond assessing general attitudes toward boredom and examines how individuals think about and regulate their own boredom experiences. This focus is essential because metacognitive beliefs critically shape maladaptive coping patterns, including the tendency toward addictive or compensatory behaviors in response to boredom (Bocci Benucci et al., 2024; Camerini et al., 2023). In the psychometric and applied domain, the MABS was developed through a mixed-methods design combining qualitative and quantitative phases and validated using advanced psychometric modeling. This approach provided robust evidence for the instrument's reliability, validity, and cross-group measurement consistency, enhancing its generalizability across diverse populations. Overall, the MABS offers a conceptually broader and psychometrically stronger framework, advancing research and applied understanding of boredom-related metacognition within behavioral science contexts.

### Theoretical implications

Research on boredom has consistently shown that it is a dual-natured phenomenon, capable of producing both adaptive and maladaptive outcomes. Individuals' beliefs about boredom are crucial in shaping this divergence (Bieleke et al., 2022; Tagliaferri et al., 2025; Westgate, 2020; Zhao et al., 2024). Building on metacognitive theory (Wells, 2000, 2009, 2019), the present study's findings extend this literature by highlighting that examining metacognitive beliefs about boredom—especially when positive and negative dimensions are analyzed separately—clarifies how boredom influences emotion, cognition, and behavior through distinct mechanisms. In this regard, the MABS conceptually bridges metacognitive theory (Wells, 2000, 2009) with emotion regulation models (Gross, 2015), suggesting that beliefs about boredom may influence both cognitive control and emotional modulation pathways. This integrative view positions boredom not merely as an affective state but as a dynamic self-regulatory process shaped by higher-order metacognitive evaluations.

For instance, P-MABS may initially activate cognitive engagement (e.g., *“Boredom helps me generate new ideas”*), whereas N-MABS tends to sustain maladaptive loops (e.g., *“Boredom leads to uncontrollable thinking spirals”*), thereby increasing vulnerability to disorders such as obsessive–compulsive disorder, generalized anxiety disorder, depression, and behavioral addictions (Cano-Lopez et al., 2022; Kim et al., 2021; Nordahl, Vollset, & Hjemdal, 2023; Spada, Caselli, Nikčević, & Wells, 2015; Tunç, Çağlın, & Ülgen, 2025). Consistent with metacognitive models, the results indicate that boredom-related metacognitive beliefs were associated with adverse emotional and behavioral outcomes. Scores of 32 or higher on the N-MABS subscale provided the best overall balance of sensitivity and specificity for distinguishing the prespecified at-risk group from community participants. While both positive and negative beliefs initially appeared relevant, only negative beliefs demonstrated meaningful risk discrimination, aligning with prior evidence (Cano-Lopez et al., 2022; Chen et al., 2021; Hua & Han, 2025; Wells, 1997; Wells & Matthews, 1996).

Beyond compensatory processes, the present findings can be more comprehensively interpreted within the I-PACE model (Brand et al., 2019). Within this framework, metacognitive beliefs about boredom may function as cognitive biases that shape both affective responses and executive control processes. More specifically, negative metacognitive beliefs (e.g., perceiving boredom as uncontrollable or harmful) may amplify negative affect and reduce perceived self-regulatory capacity, thereby increasing reliance on maladaptive coping strategies such as excessive social media use.

At the same time, positive metacognitive beliefs may contribute to self-licensing cognitions (e.g., “*I deserve to engage in social media use to cope with boredom*”), which can initially facilitate engagement through anticipated satisfaction or reward. However, over time, this satisfaction-driven engagement may transition into habitual or compulsive patterns, particularly when reinforced by intermittent rewards and reduced cognitive control. Therefore, boredom-related metacognitions may simultaneously operate through both compensatory (avoidance-driven) and satisfaction-based (approach-driven) pathways. This dual-process perspective provides a more nuanced account of how P-MABS and N-MABS contribute differentially to the development and maintenance of PSMU, aligning with contemporary integrative models of behavioral addiction.

From the perspective of the compensatory internet use theory (Kardefelt-Winther, 2014), these findings suggest that individuals may overuse social media as an attempt to escape unpleasant emotions associated with boredom. However, over time, such compensatory behaviors may reduce pleasure (Brand et al., 2019) and intensify the experience of boredom, creating a self-reinforcing loop. The results are also compatible with the components model of addiction (Griffiths, 2005), showing that negative metacognitive beliefs can activate the behavioral addiction cycle by heightening salience, worsening withdrawal, and reinforcing the use of external stimulation as a means of escape. These beliefs may reduce perceived control, intensify conflict, and elevate relapse risk. In summary, the present study elucidates the potential role of metacognitive beliefs in the dual outcomes of boredom and provides a validated instrument for further exploration of how such beliefs contribute to both adaptive and maladaptive trajectories in human behavior.

### Applied implications

The present findings offer several important implications for the assessment and intervention of PSMU. First, the MABS provides a process-oriented measure of metacognitive monitoring, capturing how individuals evaluate and regulate their boredom-related cognitions. This focus is particularly relevant given that deficits in metacognitive awareness and control have been identified as central mechanisms in behavioral addictions and problematic internet use (Brand et al., 2025; Spada et al., 2015). By quantifying both positive and negative metacognitive beliefs, the MABS allows researchers and clinicians to distinguish between adaptive and maladaptive cognitive patterns underlying PSMU.

From a clinical perspective, the empirically derived N-MABS threshold (≥32) may serve as a research-informed screening indicator to identify individuals with elevated cognitive vulnerability. Importantly, and as aforementioned, this threshold should not be interpreted as a diagnostic cut-off. Rather, it complements traditional symptom-based assessments, highlighting maladaptive beliefs that may not be fully captured in structured clinical interviews or standard psychometric scales assessing PSMU (Andreassen, Torsheim, Brunborg, & Pallesen, 2012; Casale & Flett, 2020).

In intervention contexts, these findings suggest that targeting boredom-related metacognitive beliefs may enhance treatment outcomes. For example, metacognitive therapy techniques could address dysfunctional beliefs about the uncontrollability or harm associated with boredom, while cognitive-behavioral strategies might focus on maladaptive coping, self-licensing cognitions, and habitual overuse of social media as an escape from aversive states (Kardefelt-Winther, 2014; Marino, Gini, Vieno, & Spada, 2018). Additionally, interventions could aim to strengthen adaptive metacognitive monitoring skills, enabling individuals to reinterpret boredom as a manageable and potentially constructive state rather than an aversive trigger demanding immediate avoidance (Camerini et al., 2023).

Finally, the MABS can function as a process-monitoring tool in both clinical and experimental settings. Tracking changes in N-MABS and P-MABS scores over time allows practitioners and researchers to evaluate intervention efficacy and better understand mechanisms of cognitive change. For instance, a reduction in N-MABS scores may reflect improved self-regulation and emotional flexibility, whereas increased P-MABS scores may indicate enhanced adaptive cognitive reframing. This application aligns with the broader literature linking boredom-related metacognitive beliefs to vulnerability and resilience in PSMU and related forms of problematic internet use (Brand et al., 2025; Casale & Flett, 2020; Spada et al., 2015).

Overall, the MABS offers a conceptually-grounded and empirically supported framework for bridging cognitive assessment, prevention, and intervention in behavioral addictions. By integrating metacognitive and affective dimensions, it enables a more nuanced understanding of how boredom-related beliefs contribute to vulnerability, maintenance, and potential modification of PSMU, supporting both theoretical research and applied practice.

### Strengths of the study

Beyond its theoretical contribution, the MABS demonstrates considerable applied relevance across both clinical and research contexts. It allows psychologists and researchers to systematically evaluate metacognitive beliefs about boredom, particularly within interventions addressing dysfunctional cognitive patterns, such as CBT, ACT, or metacognitive therapy. The empirically-informed threshold (approximately 32 on the N-MABS subscale) may serve as a research-based indicator of elevated vulnerability, helping practitioners identify individuals who could benefit from early preventive or resilience-enhancing strategies, especially regarding behavioral or internet-use dysregulation. The MABS also functions effectively as a process-monitoring instrument, enabling evaluation of pre–post changes in metacognitive beliefs during intervention.

A reduction in N-MABS scores may reflect improved self-regulation and emotional flexibility, whereas higher P-MABS scores may correspond to adaptive cognitive reframing and greater psychological flexibility. Moreover, the scale's specific focus on boredom-related metacognition provides a reliable and domain-sensitive instrument for examining mechanisms of change in experimental and longitudinal designs. Its use in both pre- and post-assessment frameworks could support the development of targeted treatment goals and empirical evaluation of intervention efficacy. Overall, the MABS bridges cognitive-behavioral theory with applied psychometric practice, offering a structured and empirically grounded framework for understanding how metacognitive beliefs about boredom shape both vulnerability and psychological resilience in contemporary behavioral contexts.

### Limitations and future directions

Several limitations of the present study should be acknowledged. First, the use of convenience sampling may have introduced selection bias and limited the generalizability of the findings. To address this issue, future studies should adopt random or mixed sampling strategies and employ multimethod approaches that integrate self-report data with behavioral, observational, or neurocognitive indicators (Mahudin, Cox, & Griffiths, 2012).

Second, the cross-sectional design restricted the ability to infer causal relationships or temporal dynamics among variables. In addition, test–retest reliability was examined over a relatively short two-week interval, which may not fully capture the long-term stability of the scale. Future research could therefore employ longitudinal and prospective designs to evaluate both the temporal consistency of metacognitive beliefs about boredom and their predictive role in the development, maintenance, or modification of maladaptive behaviors—particularly behavioral addictions. More specifically, future longitudinal research may employ advanced analytical frameworks such as Random-Intercept Cross-Lagged Panel Models or Latent Growth Curve Modeling to examine bidirectional associations between boredom-related metacognitions and behavioral outcomes over time. These models could clarify whether maladaptive beliefs predict later dysregulation or whether feedback loops from problematic behavior reinforce negative metacognitive patterns.

Longitudinal studies could also assess the stability and clinical relevance of the empirically derived thresholds, examining whether N-MABS scores predict relapse, treatment response, or resilience over time. Third, the exclusive use of self-report data may also introduce biases due to social desirability or subjective interpretation. Fourth, because the sample consisted solely of Iranian participants, the generalizability of the findings to other cultures or languages remains limited. Finally, the relatively small size of the at-risk subgroup may have further constrained the power of moderation and invariance analyses.

A further direction for future research involves examining the cross-contextual and cross-cultural generalizability of the MABS. Validation across different populations and clinical subgroups (e.g., individuals with depression, behavioral addictions, or anxiety disorders) would help clarify its broader applicability. Developing age-appropriate short forms for adolescents or older adults could further enhance the instrument's utility and accessibility in both clinical and community-based research.

## Conclusion

The MABS is a psychometrically robust and conceptually grounded measure for assessing how individuals think about and regulate their experiences of boredom. The present findings support its validity and applicability across both research and applied settings, highlighting its value as a domain-specific instrument that bridges cognitive, emotional, and behavioral processes. By identifying distinct patterns of positive and negative metacognitive beliefs, the MABS advances understanding of how boredom-related cognition contributes to vulnerability and self-regulation in everyday functioning and behavioral addictions. Its integration into preventive, educational, and therapeutic frameworks may offer new avenues for modifying dysfunctional beliefs and fostering adaptive emotional flexibility. By integrating metacognitive and affective dimensions, the MABS advances boredom research from a static trait toward a dynamic, self-regulatory process that reflects how individuals navigate meaning, engagement, and contemporary online challenges in everyday life. Overall, the MABS contributes to a more nuanced understanding of boredom as a metacognitive phenomenon, linking theoretical insight with practical assessment and suggesting pathways for future research on emotion regulation and behavioral health.

## Data Availability

The data are available from the first author upon reasonable request.

## Appendix A

**Table A.** Metacognitions About Boredom Scale (MABS)

Item No.	Item	1	2	3	4	5
**Positive metacognitions about boredom**						
1	Boredom helps me generate new ideas					
2	Boredom makes me more creative in problem-solving					
3	Boredom motivates me to take constructive actions					
4	Boredom helps me realign my goals and priorities					
5	Boredom increases my self-awareness and reflection					
6	Boredom allows me to focus on what truly matters					
7	Boredom makes me believe I can regulate my thoughts better					
8	Boredom is a useful signal that guides me toward growth					
**Negative metacognitions about boredom**						
9	Boredom makes me feel mentally stuck and helpless					
10	Boredom wastes my time and drains my energy					
11	Boredom leads to uncontrollable thinking spirals					
12	Boredom makes me believe there is something wrong with me					
13	Boredom feels like an uncontrollable and overwhelming state					
14	Boredom makes me feel an urgent need to escape immediately					
15	Boredom makes me vulnerable to harmful habits (e.g., overeating, excessive smartphone use)					
16	Boredom makes me lose control over my thoughts and actions					

## Appendix B Metacognitions About Boredom Scale (MABS) Development

### Step 1: Item Generation and Thematic Categorization for MABS

The development of the MABS began with a qualitative exploration aimed at identifying a full spectrum of beliefs individuals hold regarding the experience of boredom. This initial phase involved generating structured items through participant input and subsequently categorizing them thematically.

#### Data Collection

In the first phase, qualitative data were collected from 41 participants (mean age = 36.1 years, SD = 14.9; 56% female) recruited from the general community. Participants completed semi-structured face-to-face interviews in which they were invited to describe their experiences of and beliefs about boredom. Open-ended questions were used to capture a broad range of perspectives without restricting participants to predefined categories.

#### Thematic Analysis

The data were analyzed using Braun and Clarke's (2006) thematic analysis framework. The analytic process followed an inductive semantic–latent blend of thematic analysis, allowing both surface-level meanings and underlying interpretative patterns to emerge. This hybrid orientation ensured that item generation captured the experiential depth of boredom beliefs while remaining closely anchored to participants' explicit language. The procedure involved (i) familiarization with the narratives through repeated reading, (ii) generating initial codes representing distinct meanings, (iii) grouping codes into broader categories, (iv) reviewing and refining categories into coherent patterns, and (v) defining overarching themes. To ensure analytic rigor, two independent coders conducted the analysis, and discrepancies were resolved through discussion until consensus was achieved.

#### Theme Identification

Through this iterative process, two overarching themes were identified. Positive beliefs about boredom, reflecting views of it as potentially beneficial (e.g., a signal of unmet needs, a motivator of creativity, or a facilitator of reflection and growth). Negative beliefs about boredom, reflecting views of it as detrimental (e.g., emotionally aversive, cognitively impairing, or socially isolating). Each theme consisted of several subthemes that captured more specific aspects of participants' beliefs. These subthemes provided nuanced insight into the functions, meanings, and consequences attributed to boredom.

#### Item Construction

Based on the identified subthemes, raw statements from participants' narratives were paraphrased and converted into item candidates. This process followed established guidelines for item development (Boateng, Neilands, Frongillo, Melgar-Quiñonez, & Young, 2018; DeVellis, 2017). More specifically, subthemes were translated into clear and concise self-report items that captured both cognitive and evaluative dimensions of the beliefs. Overlapping or redundant statements were removed, and ambiguous wording was revised.

#### Final Item Pool

This systematic procedure resulted in a pool of 30 items representing both positive and negative beliefs about boredom. The items were distributed across the identified subthemes to ensure balanced and comprehensive coverage of the construct. This pool of items was then prepared for expert review and subsequent psychometric evaluation.

## Appendix C Rasch Model Analysis

Complementary Rasch model analyses further supported the psychometric adequacy of the scale. Mean item scores ranged from 2.11 to 4.13. Infit (0.80–1.90) and outfit (0.62–1.70) statistics generally fell within the acceptable range, indicating good item–person fit. Threshold ordering was not uniform across all items, with several instances of disordering between adjacent response categories. This pattern suggests that respondents may not have consistently distinguished between some neighboring response options. Nevertheless, the overall item-fit and reliability indices were broadly acceptable. Future studies should further examine category functioning and whether collapsing or revising adjacent response options improves measurement precision.

### Item Response Theory (IRT) Findings

Item Response Theory (IRT) analyses using the Rasch Partial Credit Model (PCM) confirmed the reliability of the scale across the latent continuum. Overall item functioning was broadly acceptable; however, threshold ordering varied across items, with several adjacent response categories showing disordering. Several threshold estimates were close to zero or disordered, suggesting limited differentiation between some adjacent response categories at particular levels of the latent trait. These deviations were minor and did not compromise overall model fit. Taken together, the results of the EFA, Rasch model, and IRT analyses provide convergent evidence for the construct validity of the scale, demonstrating a robust two-factor structure, high internal consistency, and reliable measurement precision across a wide range of ability levels.

**Table C.** Rasch model analysis (*N* = 521)

Item Response Theory (IRT)				
	Tau threshold 1	Tau threshold 2	Tau threshold 3	Tau threshold 4
Items	Strongly disagree vs. Disagree	Disagree vs. Neutral	Neutral vs. Agree	Agree vs. Strongly Agree
1	0.08	−0.25	−0.27	0.32
2	0.02	−0.14	0.15	0.24
3	0.04	−0.34	−0.31	0.53
4	−0.02	−0.37	0.19	0.13
5	−0.10	−0.21	−0.21	0.22
6	0.06	−0.42	−0.16	0.30
7	−0.07	−0.28	−0.01	0.21
8	−0.05	−0.32	0.06	0.18
9	−0.15	−0.40	0.21	0.32
10	−0.04	−0.44	0.19	−0.45
11	−0.05	−0.62	0.20	0.73
12	0.02	−0.59	0.19	0.44
13	0.02	−0.42	0.29	−0.56
14	0.05	−0.69	0.05	0.63
15	−0.01	−0.31	0.18	−0.63
16	0.01	−0.57	0.30	−0.78
